# The importance of longitudinal evaluation using DAT‐SPECT in organophosphate‐induced toxic parkinsonism

**DOI:** 10.1002/pcn5.70333

**Published:** 2026-04-29

**Authors:** Shuhei Nakao, Yoichiro Kogoh, Hideki Funahashi, Yusei Yanoma, Minako Azuma, Yasushi Ishida, Yoji Hirano

**Affiliations:** ^1^ Department of Psychiatry, Division of Clinical Neuroscience, Faculty of Medicine University of Miyazaki Miyazaki Japan; ^2^ Miyazaki Prefectural Miyazaki Hospital Miyazaki Japan; ^3^ Department of Radiology, Division of Pathophysiological Diagnosis and Therapy, Faculty of Medicine University of Miyazaki Miyazaki Japan; ^4^ Nozaki Hospital Miyazaki Japan; ^5^ Institute of Industrial Science The University of Tokyo Tokyo Japan

**Keywords:** amantadine, DAT‐SPECT, organophosphate poisoning, organophosphates, toxic parkinsonism, trihexyphenidyl

## Abstract

**Background:**

Clinicians encounter considerable challenges in the diagnosis and treatment of organophosphate‐induced toxic parkinsonism following acute poisoning. Here, we report the importance of longitudinal evaluation of a patient with organophosphate‐induced toxic parkinsonism using dopamine transporter single‐photon emission computed tomography (DAT‐SPECT). This case may provide crucial insights into the pathophysiology of the syndrome and inform therapeutic decision‐making.

**Case Presentation:**

We report a case of organophosphate‐induced toxic parkinsonism with persistent parkinsonian symptoms evaluated longitudinally using DAT‐SPECT. A 68‐year‐old woman with a history of bipolar disorder was found collapsed at home on Day 0 with impaired consciousness, miosis, hypersalivation, and bradycardia. Organophosphate poisoning was diagnosed based on markedly reduced cholinesterase levels and the presence of an organophosphate pesticide in a shed at her home. Parkinsonian symptoms gradually emerged from Day 18, including hypophonia and a gait disturbance. On Day 60, DAT‐SPECT showed reduced radiotracer uptake in the bilateral striatum, more pronounced on the right. After toxic parkinsonism was diagnosed, l‐dopa was titrated up to 600 mg/day without clinical benefit and discontinued due to adverse effects. Treatment with amantadine and trihexyphenidyl was then initiated, resulting in partial symptomatic improvement, although symptoms persisted. Despite clinical improvement, follow‐up DAT‐SPECT on Day 131 showed a further reduction in striatal tracer uptake, whereas quantitative improvement was observed on Day 200.

**Conclusion:**

Parkinsonism is an uncommon sequela of organophosphate poisoning and may be accompanied by abnormal DAT‐SPECT findings. Moreover, amantadine and trihexyphenidyl may represent effective therapeutic options for this condition.

## Graphical abstract

We performed longitudinal evaluation using magnetic resonance imaging (MRI) and dopamine transporter single‐photon emission computed tomography (DAT‐SPECT) in a case of organophosphate‐induced toxic parkinsonism. Fluid‐attenuated inversion recovery (FLAIR) images showed bilateral striatal hyperintensity in the acute phase, followed by reduction of signal abnormalities with progressive striatal volume loss and enlargement of the anterior horns of the lateral ventricles. In contrast, DAT‐SPECT demonstrated an initial reduction in striatal uptake, further decline, and subsequent partial recovery over time. Partial clinical improvement was observed during treatment with amantadine and trihexyphenidyl.

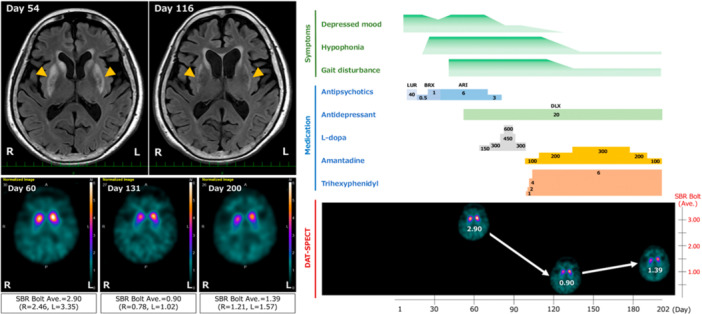

## INTRODUCTION

Suicide remains a major global public health concern, accounting for over 700,000 deaths annually and ranking among the leading causes of mortality.^1^ Despite advances in mental health care, many countries continue to experience substantial suicide burdens.^1^, ^2^ In Japan, suicide rates declined in the early 2010s following national prevention efforts but have recently stagnated or increased in certain populations, including young people and some older adults.^3^ In addition, patterns of suicide and the methods employed differ between urban and rural regions, underscoring the necessity for region‐specific and context‐sensitive prevention strategies.^4^, ^5^

Organophosphates are widely used as insecticides and chemical warfare agents such as sarin. Exposure occurs through chronic occupational contact or acute poisoning via suicide attempts, accidental ingestion, or warfare. The primary toxic mechanism is acetylcholinesterase inhibition.^6^ Toxic parkinsonism is a rare complication, presenting with symptoms similar to idiopathic Parkinson's disease (PD), including resting tremor, rigidity, and bradykinesia, but typically showing poor responsiveness to l‐dopa.^7^ Symptom onset is typically delayed, occurring days to weeks after exposure.^8^, ^9^, ^10^ Neuroimaging usually reveals no specific abnormalities on magnetic resonance imaging (MRI), although T2‐weighted MRI may show high signal intensity in the putamen and caudate nucleus in some cases,^11^ and hyperperfusion of the putamen and thalamus has also been reported on *N*‐isopropyl‐*p*‐[^123^I]‐iodoamphetamine single‐photon emission computed tomography (^123^I‐IMP SPECT).^9^ Furthermore, Aubeneau et al. (2008) described a case that developed parkinsonism after 10 years of chronic organophosphate exposure, in which dopamine transporter‐SPECT (DAT‐SPECT) demonstrated loss of presynaptic function.^12^ To date, most reports of organophosphate‐induced toxic parkinsonism are isolated case reports, and the condition is considered rare. While spontaneous recovery has been documented in some instances,^7^ the present case exhibited persistent parkinsonian symptoms even after 2 years. Although previous studies have described longitudinal changes in brain function using cerebral blood flow SPECT,^9^ to the best of our knowledge, no study has reported serial changes using DAT‐SPECT in this condition.

## CASE PRESENTATION

The patient was a 68‐year‐old Japanese female with no notable medical or family history of psychiatric or neurodegenerative disorders. She completed high school and worked in food packaging and later in her family's landscaping and farming businesses, raising four children. She had a transient depressive episode in her mid‐30s that resolved spontaneously.

At age 50, depressive symptoms recurred, and she was diagnosed with bipolar disorder. Initial treatment with paroxetine was ineffective. Lithium and olanzapine were discontinued due to adverse effects, and she was stabilized on lurasidone (60 mg/day), ethyl loflazepate (1 mg/day), and lamotrigine (300 mg/day).

On Day 0, she was found collapsed in her bathroom at 09:15, approximately 15 min after speaking with her family. She presented with severely impaired consciousness, miosis, hypersalivation, and bradycardia. She was intubated due to impaired consciousness and the risk of airway obstruction from excessive secretions and transported by air ambulance. Oxygen saturation remained ≥80% after initial contact with emergency services at 09:31 and improved to 100% following intubation at 10:01. Arterial blood gas analysis on arrival at 11:00 showed no evidence of hypoxemia. Initial head computed tomography (CT) showed no abnormalities, while laboratory testing revealed markedly reduced serum cholinesterase (41 U/L). A container of organophosphate pesticide labeled as containing fenthion was discovered in a shed at her home, confirming acute organophosphate poisoning. She was treated with pralidoxime methiodide and admitted to the emergency intensive care unit. On Day 8, after medical stabilization, she was transferred to our psychiatric ward.

**Figure 1:**
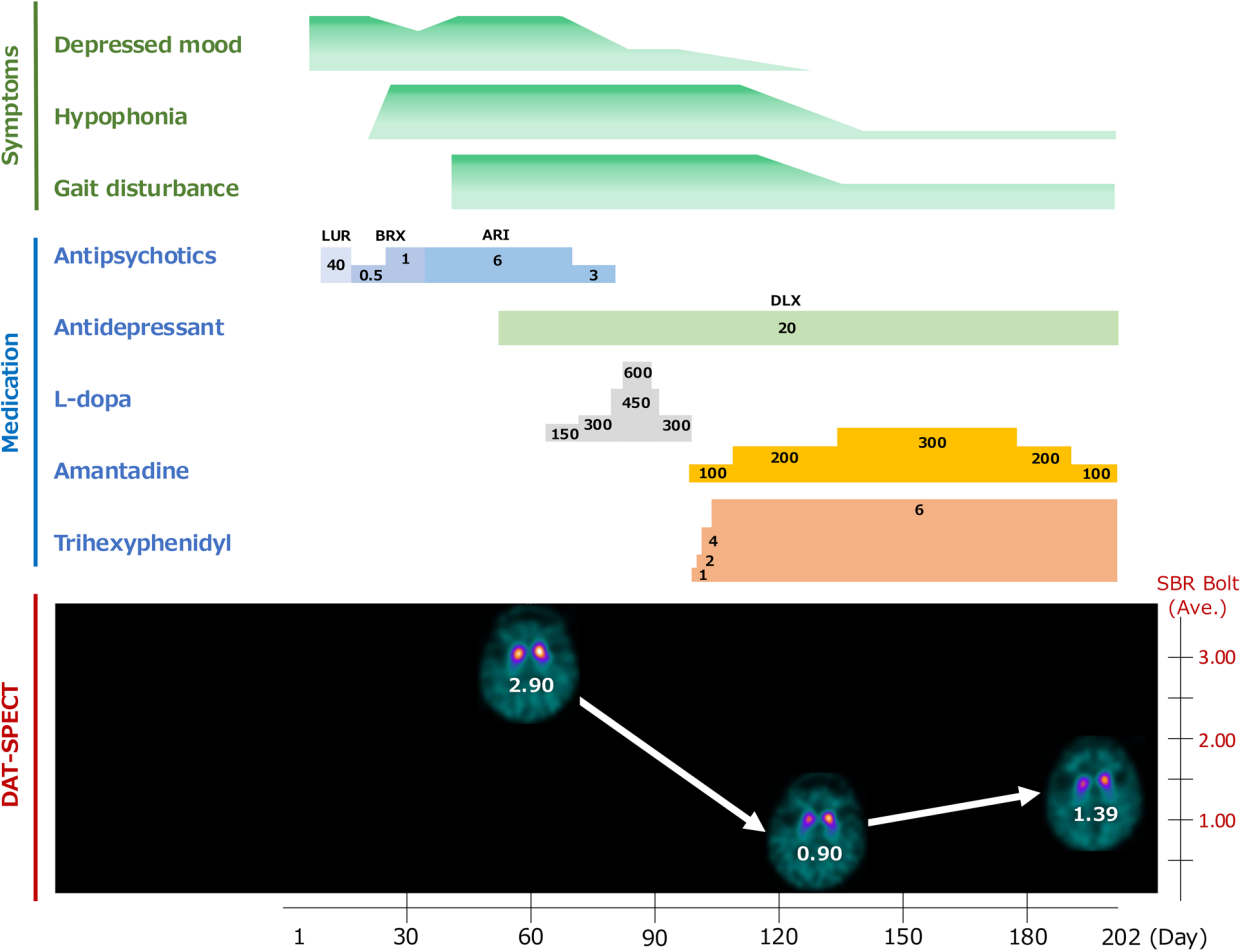
Clinical course of the patient diagnosed with organophosphate‐induced toxic parkinsonism. Abbreviations: ARI, aripiprazole; BRX, brexpiprazole; DAT‐SPECT, dopamine transporter single‐photon emission computed tomography; DLX, duloxetine (medication dose: mg/day); LUR, lurasidone; SBR_Bolt_, specific binding ratio values using the Tossici‐Bolt method.

A detailed clinical course is shown in Figure 1. At the time of ward transfer, she exhibited disorientation, depressive mood, psychomotor retardation, and both grandiose and nihilistic delusions, consistent with a mixed affective state. Although bedridden due to disuse, her voice remained preserved, and no parkinsonian features were initially observed. Lurasidone and ethyl loflazepate were reinstated, but psychiatric symptoms remained refractory. Brexpiprazole was initiated (titrated to 1 mg/day), but on Day 18, she developed sudden hypophonia. Laryngoscopy was unremarkable, and the symptom was initially attributed to reduced motivation. Brexpiprazole was replaced with aripiprazole (6 mg/day) on Day 36. Although mood and activities of daily living (ADL) gradually improved, hypophonia persisted. On Day 38, parkinsonian signs emerged, including resting tremor of the upper limbs without clear lateral asymmetry, freezing of gait, shuffling steps, blepharoptosis, and left‐dominant rigidity. Given concurrent antipsychotic use, drug‐induced parkinsonism and syndromes such as Dementia with Lewy Bodies (DLB) were considered. Mini‐Mental State Examination (MMSE) and Hasegawa's Dementia Scale‐Revised (HDS‐R) scores were both 25/30, with deficits in orientation, serial 7s, and recall. The pareidolia test revealed an illusion rate of 27.5%.

**Figure 2:**
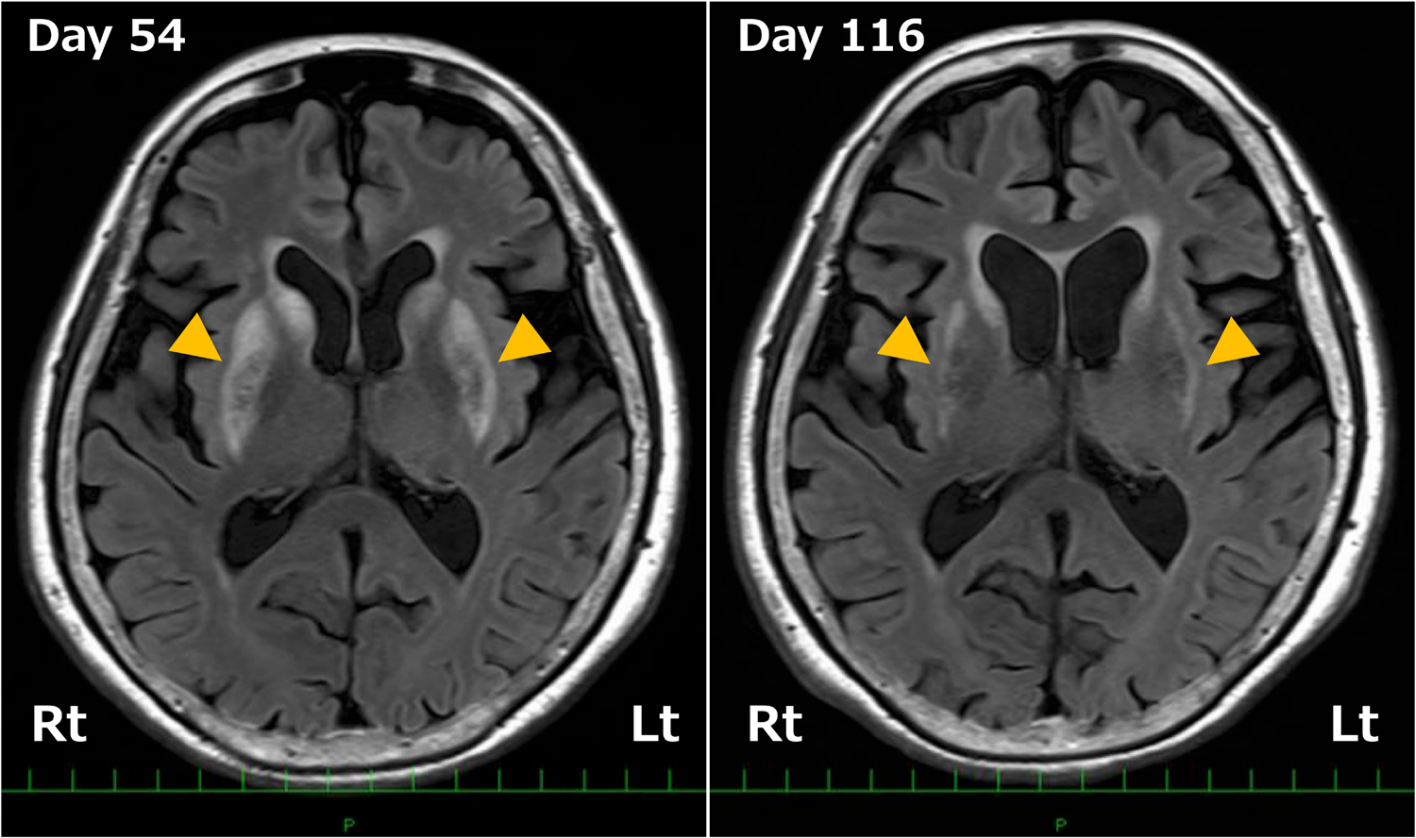
Longitudinal T2‐weighted fluid‐attenuated inversion recovery (FLAIR) axial images of the present case. On Day 54, bilateral hyperintense lesions in the striatum (indicated by the orange triangles) are observed (left image). On Day 116, decreased bilateral striatal hyperintensity and bilateral striatal volume loss (indicated by the orange triangles), with enlargement of the anterior horns of the lateral ventricles, are observed (right image).

**Figure 3:**
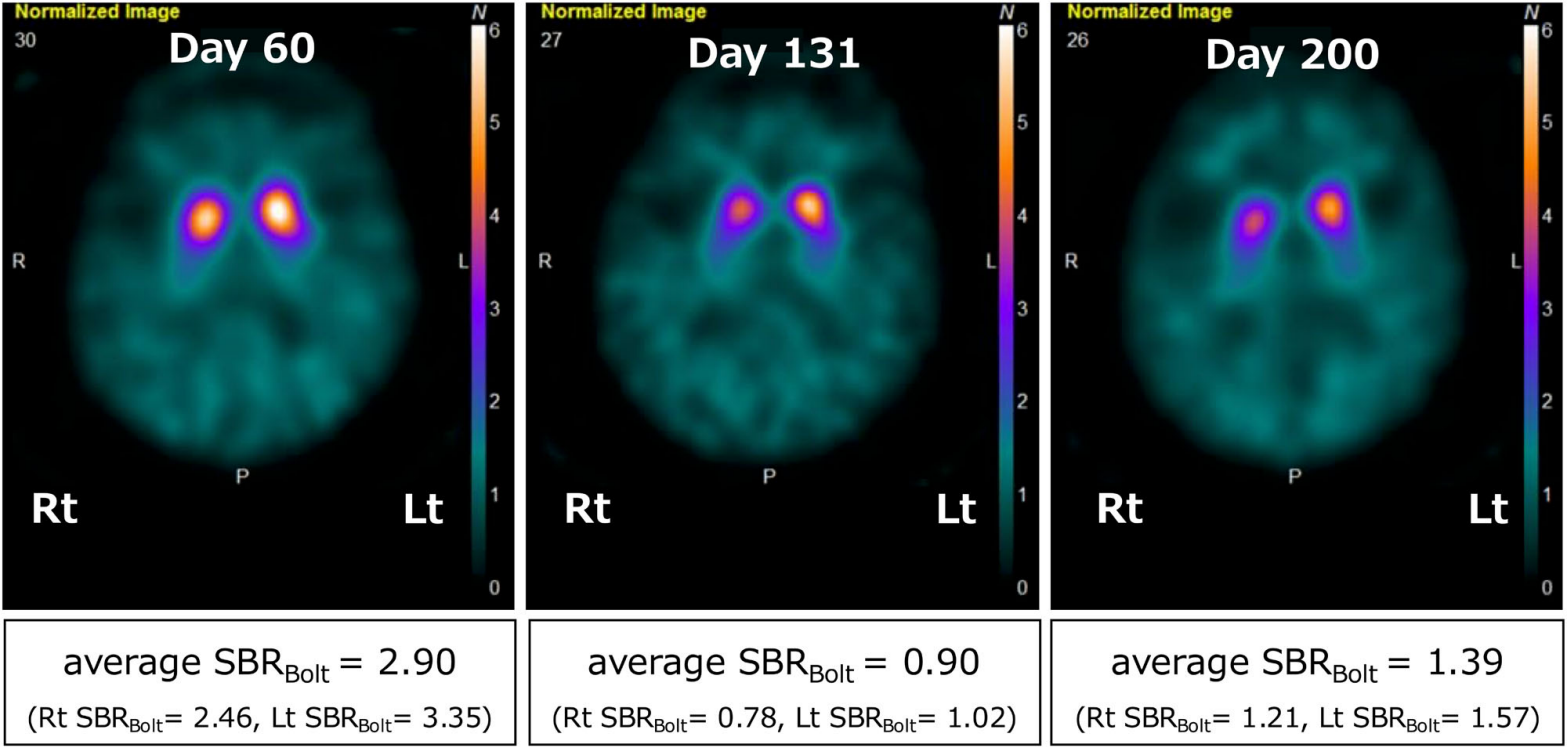
Dopamine transporter single‐photon emission computed tomography (DAT‐SPECT) images of the present case. The specific binding ratio (SBR) values were obtained using the Tossici‐Bolt method (SBR_Bolt_). On Day 60, bilateral striatal uptake is reduced, with an average SBR_Bolt_ of 2.90 (left image). On Day 131, further reduction in striatal uptake is observed, with an average SBR_Bolt_ of 0.90 (middle image). On Day 200, partial recovery of striatal uptake is observed, with an average SBR_Bolt_ of 1.39 (right image).

To evaluate potential structural abnormalities of the brain, detailed MRI examinations were conducted. T1‐ and T2‐weighted images, T2‐weighted fluid‐attenuated inversion recovery (FLAIR) images, and diffusion‐weighted imaging (DWI) on Day 54 demonstrated high‐signal lesions in the bilateral striatum (Figure 2), indicative of structural damage consistent with organophosphate toxicity. Cerebral blood flow SPECT showed relative hypoperfusion in the bilateral occipital and frontal lobes. The specific binding ratio (SBR) for DAT‐SPECT was quantified using the Tossici‐Bolt method (SBR_Bolt_). DAT‐SPECT on Day 60 revealed bilateral striatal uptake reduction, more pronounced on the right (Rt SBR_Bolt_ = 2.46; Lt SBR_Bolt_ = 3.35), with an average SBR_Bolt_ of 2.90 (Figure 3). Myocardial ^123^I‐metaiodobenzylguanidine (MIBG) scintigraphy was unremarkable. Electroencephalogram (EEG) showed dominant beta activity (around 20 Hz) without epileptiform discharges, likely related to ethyl loflazepate.

Based on the clinical and imaging findings, together with the delayed onset of parkinsonism following confirmed organophosphate poisoning, she was diagnosed with organophosphate‐induced toxic parkinsonism. l‐Dopa was titrated up to 600 mg/day, and no clear symptomatic improvement was observed during this period. However, treatment was discontinued because the patient developed visual illusions suggestive of dopaminergic adverse effects before its efficacy could be adequately evaluated. Aripiprazole was discontinued, and duloxetine (20 mg/day) was initiated to address residual affective symptoms, which subsequently improved. Amantadine was started on Day 101 at 100 mg/day, resulting in improvements in hypophonia and gait. Trihexyphenidyl was added on Day 117 at 2 mg/day, providing additional symptomatic benefit. Dosages were increased to amantadine 300 mg/day and trihexyphenidyl 6 mg/day. Her voice volume returned to an audible level, and she regained the ability to ambulate outdoors with supervision (Figure 1).

Follow‐up MRI on Day 116 showed decreased striatal hyperintensity on T2‐weighted FLAIR images, reduced striatal volume on T1‐ and T2‐weighted images, and normalization of diffusion signals on DWI (Figure 2). DAT‐SPECT on Day 131 demonstrated further decline in striatal uptake (average SBR_Bolt_ = 0.90) (Figure 3) despite clinical improvement (Figure 1). However, repeat DAT‐SPECT on Day 200 showed partial recovery (average SBR_Bolt_ = 1.39) (Figures 1 and 3), and EEG on Day 191 revealed slight increases in alpha activity and reductions in beta activity.

## DISCUSSION

### Pathophysiology of organophosphate‐induced toxic parkinsonism

Exposure to organophosphates can cause mitochondrial depletion and morphological disruption. Impaired axonal mitochondrial transport may reduce energy supply and calcium regulation at nerve terminals, resulting in decreased ATP production, increased oxidative stress from reactive oxygen species (ROS), and enhanced apoptosis.^13^, ^14^, ^15^, ^16^ The nigrostriatal system is particularly susceptible to ROS because dopamine metabolism generates substantial oxidative load and the region contains abundant iron, which amplifies ROS production.^13^, ^17^ Oxidative stress following organophosphate exposure may activate microglia and astrocytes, leading to neuronal damage through proinflammatory cytokines such as IL‐1β, TNF‐α, and IL‐6, as well as nitric oxide.^18^, ^19^, ^20^, ^21^, ^22^ In this case, MRI revealed high‐signal abnormalities in the striatum, suggesting structural damage. DAT‐SPECT demonstrated bilateral reductions in striatal DAT binding, indicating presynaptic dopaminergic impairment. Preserved myocardial MIBG scintigraphy findings further support that this abnormality was unlikely to reflect PD‐type neurodegeneration, consistent with the gradual clinical improvement observed over time.

A potential limitation is that hypoxic encephalopathy cannot be completely excluded, as the patient required intubation at presentation. However, the clinical course suggests that severe hypoxic encephalopathy was unlikely. The interval between the last known well time and discovery was approximately 15 min, and oxygen saturation remained ≥80% after initial contact with emergency services, improving to 100% following intubation. Arterial blood gas analysis on arrival showed no evidence of hypoxemia, and intubation was performed due to impaired consciousness and the risk of airway obstruction from excessive secretions, rather than respiratory failure. While hypoxic encephalopathy cannot be entirely ruled out, organophosphate‐induced neurotoxicity is considered the primary mechanism underlying the observed neurological findings.

### Differentiating organophosphate‐induced toxic parkinsonism from Parkinson's disease and drug‐induced parkinsonism

Whereas DAT‐SPECT generally reflects the integrity of presynaptic dopaminergic terminal density,^23^ non‐degenerative parkinsonian syndromes—such as drug‐induced parkinsonism—typically do not exhibit reduced DAT binding.^24^, ^25^, ^26^ In contrast, organophosphate‐induced parkinsonism may, in certain cases, entail impairment at the level of presynaptic dopaminergic terminals. Thus, the presence of reduced striatal DAT uptake on DAT‐SPECT may facilitate the differential diagnosis between organophosphate‐induced toxic parkinsonism and drug‐induced parkinsonism. However, both organophosphate‐induced toxic parkinsonism and PD may exhibit reduced striatal uptake on DAT‐SPECT,^27^ rendering differentiation between the two conditions challenging when relying solely on DAT‐SPECT findings. Therefore, accurate distinction should be grounded in a comprehensive assessment of the mode of onset, clinical features, and myocardial MIBG scintigraphy findings. In general, PD typically shows reduced myocardial MIBG uptake, reflecting cardiac sympathetic denervation.^28^

### Clinical implications and lessons from this case

#### Dissociation between clinical symptoms, MRI, and DAT‐SPECT findings

FLAIR hyperintensity in the striatum decreased over time, while striatal volume loss and enlargement of the anterior horns of the lateral ventricles were observed. During the same period, clinical improvement remained limited. In addition, MRI revealed no evident lateral asymmetry, whereas DAT‐SPECT demonstrated progressively increasing asymmetry over time. This dissociation indicates that structural changes on MRI, longitudinal changes in DAT‐SPECT findings, and clinical manifestations represent distinct aspects of the disease process and do not necessarily progress in parallel. It should be noted that, under ongoing pharmacological modulation with amantadine and trihexyphenidyl, the relationship between these neuroimaging findings and clinical symptoms becomes difficult to interpret, and a direct one‐to‐one correspondence between imaging changes and clinical recovery cannot be reliably inferred.

#### Possible reasons for onset and prolonged parkinsonism in this case

Parkinsonism attributable to organophosphate poisoning typically improves within several weeks to 2 months.^9^ However, in this case, symptoms persisted for at least 2 years. Although this case represents secondary parkinsonism following acute intoxication, prior studies have examined associations between chronic exposure to organophosphate‐containing pesticides and an elevated risk of chronic neurodegenerative diseases such as PD.^29^, ^30^ Moreover, a study conducted in rural California—where organophosphate pesticide exposure is presumed—identified associations between paraoxonase 1 (PON1) gene polymorphisms and PD susceptibility.^17^ Because genetic testing was not performed in this case, the presence of underlying genetic vulnerability remains undetermined. There was no documented family history of PD. Although the patient was engaged in agriculture and may have experienced chronic occupational exposure to organophosphate, the precise cause of the unusually prolonged persistence of symptoms cannot be conclusively established.

#### Therapeutic efficacy of amantadine and trihexyphenidyl

In this case, amantadine and trihexyphenidyl were associated with partial symptomatic improvement, suggesting that these agents may contribute to symptom management in organophosphate‐induced toxic parkinsonism. Amantadine is widely regarded as exerting its antiparkinsonian effects primarily through antagonism of *N*‐methyl‐d‐aspartate (NMDA) receptors, thereby modulating glutamatergic hyperactivity within the basal ganglia circuits.^31^ Trihexyphenidyl, in contrast, is considered to improve parkinsonian symptoms by attenuating striatal cholinergic activity, which results in restoration of the dopaminergic–cholinergic balance within the basal ganglia circuits.^32^

## CONCLUSION

Against the backdrop of persistent global and region‐specific suicide burdens, organophosphate exposure remains a relevant public health concern. This case demonstrates impairment at the level of presynaptic dopaminergic terminals, providing novel insight into the pathophysiology of organophosphate‐induced toxic parkinsonism. Serial DAT‐SPECT findings extend previous isolated reports and go beyond structural or perfusion imaging by directly capturing dynamic dopaminergic dysfunction and subsequent partial recovery. In addition, the observed partial therapeutic response to amantadine and trihexyphenidyl has practical implications for the management of this otherwise poorly characterized condition.

## AUTHOR CONTRIBUTIONS

Shuhei Nakao, Yusei Yanoma, and Yoichiro Kogoh treated the patient. Shuhei Nakao wrote the first draft. Hideki Funahashi, Yoichiro Kogoh, Minako Azuma, Yasushi Ishida, and Yoji Hirano critically revised the manuscript. All authors have read and approved the final manuscript.

## CONFLICT OF INTEREST STATEMENT

The authors declare no conflicts of interest.

## ETHICS APPROVAL STATEMENT

Approval of the research protocol by an institutional review board: N/A. Animal studies: N/A.

## PATIENT CONSENT STATEMENT

The patient and her family provided informed consent for the publication of this case report.

## CLINICAL TRIAL REGISTRATION

N/A.

## Data Availability

Data sharing is not applicable to this article as no datasets were generated or analyzed during the current study.
